# Supplementary material for: Psychosocial, cultural, and academic challenges to Saudi Arabian students in Australia
Source: PLoS One. 2022 Jan 31;17(1):e0262585. doi: 10.1371/journal.pone.0262585 (PMC8803179; doi:10.1371/journal.pone.0262585)
Supplement: Attachment — Submitted filename: Response to reviewers Comments.docx [file pone.0262585.s001.docx]

**Response to Reviewers Comments**

Additional Academic Editor Comments:

1. Why was ethical approval for the present study obtained from the Ethical Review Committee of the University of Faisalabad based in Pakistan while the study was funded by King Abdulaziz University based in Saudi Arabia? The first author is based in Saudi Arabia. Why wasn’t ethical approval obtained from King Abdulaziz University, Saudi Arabia?

Response:

Initially we planned to execute this study for the international students of two Muslim countries i.e., Pakistan and Saudi Arabia. The original idea was from one of the authors from Pakistan, but we got very poor response from Pakistani students and moreover the scholarships are not being well organized in Pakistan as compared to Saudi Arabia. Luckily Saudi Arabian students responded to our project overwhelmingly, so we decided to limit ourselves to the Saudi Arabian students. Changes were carried out in the title and in other aspects.

2.Keywords: Add ‘’medical student’’.

Response:

Response: Added as suggested.

3. Introduction-2nd line: Replace ‘’SA” with ‘’Saudi Arabia (SA)”. SA is used as an abbreviation for the first time in the main text here.

Response: Added Saudi Arabia

4.Introduction-1st paragraph-Last line: There is no mention of ‘’Kingdom’’ earlier in the paragraph.

Response: The word kingdom has been added in the start of the introduction.

5.Introduction-2nd paragraph-1st sentence: Provide a reference to support this statement at the end of the sentence.

Response: Sentence modified and reference has been added.

6.Introduction-2nd paragraph-5th line: Embracing or embarrassing?

Response: Its embarrassing, the word has been corrected.

7.Introduction-2nd paragraph-5th line: How is it embarrassing? Provide details rather than a blanket statement.

Response: Sentence has been modified.

8.Introduction-2nd paragraph-Last 2 sentences: The ‘’connect……’’ is missing with the rest of the paragraph. Moreover, the last sentence lacks clarity.

Response: Both sentences have been modified.

9. Introduction-3rd paragraph-6th line: Good universities?

Response: Corrected

10. Introduction-Last paragraph-7th line: Mention who are you referring to in relation to ‘’experiences and learnt lessons’’.

Response: The sentence has been modified.

11.Introduction-Last paragraph-8th line: Delete ‘’was’’.

Response: Done

12. Introduction: You have referred to the ‘’Australian culture’’ in the conclusion section of the text. Although the Australian culture is briefly touched upon in the introduction section, strengthen the introduction to the Australian culture in the introduction section.

Response: Elaborated in initial lines of 2 paragraph of introduction section.

13. Methods: Specify the name of the institution from where the ethical approval was obtained.

Response: Name of the institution has been mentioned in M&M

14.Methods: What do you mean by “Students’ consent was taken, and filling the questionnaire was also considered their consent to participate in the study.”? Was the first consent obtained on-line as well? How was it obtained? This sentence needs to be clarified and accordingly revised. It is not clear regarding how exactly consent was obtained.

Response: Reshaped the sentence of student’s consent.

15.	Methods: What do you mean by ‘’religious rights wish to stay further in Australia”?

Response: Reshaped the sentence for clarity
16.	Methods: Provide a reference for ‘’five-point Likert scale’’.

Response: Added a reference for five point Likert scale methodology
17.	Methods: Provide a reference for ‘’SPSS’’.

Response: A reference was added for SPSS methodology

18.	Methods: The methods section requires further work. Reorganize the methods section into relevant categories (example: setting/design, sample/participants, procedure/instruments, analysis, ethical approval).

Response: Rewritten the “methods” section as suggested.

a. Provide further details as to how the questionnaire was developed.

Response:

The details have been added. We used LimeSurvey service for conducting online survey Survey Link: [https://nsbstat.com/sms/index.php/296395?newtest=Y&lang=en#](https://nsbstat.com/sms/index.php/296395?newtest=Y&lang=en)

b. Mention further details regarding when the study was conducted. The title suggests that the participants were medical students (Saudi Arabian medical students studying in Australia), but no mention of medical students in the methods section.

Response: Modified as per recommendation.

We invited only those Saudi students registered for an undergraduate medical degree, medical graduates at various institutes in Australia for postgraduation, and Ph.D. in different medical sciences disciplines. This has been added in the methods.

c. How were the participants recruited? How was the sample size calculated?

Response:

The sample size calculation and details of participants recruitment have been added.

d. How was the questionnaire distributed online? Provide description of your sample. Two hundred and nineteen (219) Saudi Arabian students studying in Australia participated in the present study (1st line of the results section).

Response: This explanation has been added in methods section

e. What was the total number of Saudi Arabian medical students studying in Australia at the time of conducting the survey?

Response: There were 450 Saudi students registered for an undergraduate medical degree, medical graduates at various institutes in Australia for postgraduation, and Ph.D. in different medical sciences disciplines at the time of our study. This has been added in the methods.

f. What was the response rate? It is important to state the response rate of the survey (219 of how many sent). Questionnaires were sent to how many (potential participants) in total.

Response: Of the total 450, 219 students responded to our questionnaire, so the response rate was 49%.

g. The percentage of people who responded is an important criterion to judge the statistics.

Response: This has been included in the methods section

h. Was there any pattern to those not responding?

Response: we, exactly don’t know.

i. How likely is it that the composition of all the people to whom the questionnaire was sent, is significantly different from those who responded to the questionnaire?

Response: It is less likely that the composition of our respondents was different from the non-respondents because, in our study, there were respondents from both genders, married and unmarried, and from all three groups (undergraduate medical degree, medical graduates doing postgraduation, and Ph.D. in different medical sciences disciplines).

19.Results: Thirty-nine participants (39) were pursuing a PhD. Did you consider even the PhD students as medical students?

Response:

Thank you for pointing out this. We have corrected this throughout the manuscript. We invited students who were registered there for undergraduate medical degree, medical graduate who were in Australia for postgraduation and for Ph.D. in different medical sciences disciplines. This has been added in the methods.

20.	Results-Table 1: What do you mean by ‘’completed course/s in Australia’’? Needs further elaboration.

Response: These are the students who completed their bachelor’s degree and enrolled in master degree or completed master degree and enrolled in Ph.D. and also included those who were performing internship.

21.	Results-Table 1: It would be more interesting to relate the ‘’number of visits to Saudi Arabia after arrival in Australia’’ with the duration of stay in Australia. How many days were spent in Saudi Arabia during these visits

Response: We are sorry now this question was not included in our questionnaire so we are unable to discuss this point.

22.Results-Financier of your study: What is the criterion to differentiate ‘’Saudi government’’ from ‘’University scholarship’’? Aren’t most of the universities in Saudi Arabia run by the government. Provide details to avoid further questions and accordingly revise the categories. I would like to know what ‘’others’’ stands for.

Response:

“Others” stand for all private and nonprofit organizations through which international scholarship can be obtained.

There are multiple programs for motivating the Saudi Arabian students for higher studies in multiple overseas venues. Many Governmental, private organizations & public Universities offer scholar ship for deserving students. We are citing two links. In first link there are details of Saudi Arabian scholarship agencies and in second one the foreign Universities themselves offer scholarships to foreign students.

a. KSA Government Scholarship Program • King Abdullah Scholarship Program (KASP) • Custodian of the Two Holy Mosques Scholarship Program • King Salman Scholarship Program King Abdulaziz & His Companions Foundation for Giftedness & Creativity (Mawhiba) Royal Commission of Al-Ula Scholarship Program KAUST Gifted Student Program MiSK Foundation Fellowship & Traineeship Technical and Vocational Training Corporation (TVTC) Institute of Public Administration (IPA) - ARAMCO - SABIC - Other Saudi universities.

Link: <https://educationusa.state.gov/scholarships/scholarships-available-saudi-students>.

b. King Faisal Foundation scholarships for international KSA students.

[King Faisal Foundation (KFF) (bibalex.org)](https://www.bibalex.org/he_funding/donors/Details.aspx?ID=112)

c. The Australian Government has a large scholarship program for international students and invests some $200 million dollars per year in programs at universities and other tertiary institutions around the country. Many of the country’s top institutions including The University of Melbourne, The University of Sydney, and the Australian National University (ANU) offer scholarships to study in Australia with added extras, like health cover. Link: [Scholarships for International Students to Study Abroad 2020-2021 | IDP Saudi Arabia](https://www.idp.com/saudiarabia/scholarships/?lang=en)

23.Results-Table 1: Should it be ‘’other degree’’ or ‘’other courses’’ (completed course/s in Australia)?

Response: Yes, you are right. The right word will be “course”

24.Results: What is the age of the participants?

Response: Details of age of our participants have been added in the result section. We are sorry for omission.

25.Discussion-1st sentence: Are you referring to ‘’Saudi Arabian medical students’’?

Response: We have rephrased the sentence.

26.Discussion-2nd paragraph-1st sentence: “Early marriage is part of Saudi Arabian culture, so most of our study participants (70%) were married ………. ‘’. You haven’t reported the age of the participants to begin with and therefore how could you provide this reason for most of the study participants being married. It appears to me that most of the participants are married because most of them are postgraduate students or pursuing a PhD; only 13 participants were undergraduate students.

Response: Age of the respondents has been added in table 1. However, you are right. So, we have modified the sentence.

27. Discussion-2nd paragraph-Last sentence: Revise ‘’previous study by Midgley W. (2009)’’.

Response: Revised as suggested.

28. Discussion-2nd paragraph-Last sentence: Who were the Saudi students in the previous study? Background/course pursuing?

Response: This has been explained in detail. Those Saudi Students were undergoing training for ELICOS (English Language Intensive Course for Overseas Students).

29.Discussion-Psychological aspects-1st paragraph: Provide a reference at the end of the 3rd sentence (4th line).

Response: A reference has been added.

30.Discussion-Cultural aspects-2nd line: State the religious activities.

Response: The sentence has been rephrased.

31.Discussion-Cultural aspects: Were questions on ‘’halal’’ food included in the questionnaire?

Response: No, we didn’t ask directly but majority of Muslims eat at the places where Halal food is available. And this facility is available and mentioned in all the restaurants in almost all cities of Australia

32.	Discussion-Financial aspects: NGOs is mentioned here. If I am not mistaken, there is no mention of NGOs in the results section.

Response: We have included this in the results.

33.	Discussion-Limitations of the study: The aim/objective of the study revolved around medical students. Isn’t it? Then why mention that your study related to only medical students. I vehemently do not agree with the related limitations of the study mentioned. Secondly, do not provide a blanket statement that the online nature of the study was a limitation. In what way was the online nature of the study a limitation. What were the limitations that you perceived/faced due to the online nature of the study?

Response: Thanks for the guidance. We’ve rewritten this section.

We have included the following disadvantages of the online surveys. Online surveys have several drawbacks, including poor response rate, and it is hard to ascertain whether the right person completed the questionnaire. It's difficult to contact people who don't use social media, and frequent requests to finish the survey can be frustrating for participants and often backfire.

34.Discussion: Rewrite the limitations of the study.

Response: The limitations of the study have been rewritten.

35.Discussion: Provide a separate paragraph on the implications of the study.

Response: A separate paragraph has been added as advised.

36.Discussion: Provide a separate paragraph on recommendations/future directions.

Response: Future recommendations have been mentioned separately.
37.Conclusions: Draft a better paragraph on the conclusions of the study. Make sure that the conclusions drawn are based on your observations (data) and results while doing so.

Response: Rephrased conclusions with elaboration in light of our findings

**Comments to the Author**

1. Is the manuscript technically sound, and do the data support the conclusions?

The manuscript must describe a technically sound piece of scientific research with data that supports the conclusions. Experiments must have been conducted rigorously, with appropriate controls, replication, and sample sizes. The conclusions must be drawn appropriately based on the data presented.

Reviewer #1: Yes

Reviewer #2: Yes

2. Has the statistical analysis been performed appropriately and rigorously?

Reviewer #1: Yes

Reviewer #2: Yes

3. Have the authors made all data underlying the findings in their manuscript fully available?

The [PLOS Data policy](http://www.plosone.org/static/policies.action#sharing) requires authors to make all data underlying the findings described in their manuscript fully available without restriction, with rare exception (please refer to the Data Availability Statement in the manuscript PDF file). The data should be provided as part of the manuscript or its supporting information, or deposited to a public repository. For example, in addition to summary statistics, the data points behind means, medians and variance measures should be available. If there are restrictions on publicly sharing data—e.g. participant privacy or use of data from a third party—those must be specified.

 Reviewer #1: Yes

Reviewer #2: Yes

4. Is the manuscript presented in an intelligible fashion and written in standard English?

PLOS ONE does not copyedit accepted manuscripts, so the language in submitted articles must be clear, correct, and unambiguous. Any typographical or grammatical errors should be corrected at revision, so please note any specific errors here.

 Reviewer #1: Yes

Reviewer #2: No

 5. Review Comments to the Author

Please use the space provided to explain your answers to the questions above. You may also include additional comments for the author, including concerns about dual publication, research ethics, or publication ethics. (Please upload your review as an attachment if it exceeds 20,000 characters)

Reviewer #1: The research is well-written and focused on medical students with extensive details. The students sample is mainly postgraduate, in which we expect more fluency in English language in addition to more likely financial and social independence. I think this study does not represent undergraduate students.

Response: Yes, you are right. The observation is really nice. We invited only those Saudi students registered for an undergraduate medical degree, medical graduates at various institutes in Australia for postgraduation, and Ph.D. in different medical sciences disciplines. This has been added in the methods.

There are a few grammar conflicts need to be addressed such as:
Page 2: Objectives should not be in the past tense.

Response: Thanks for pointing it out. Corrected

Page 3: "In the case of interactive lectures, the situation becomes rather embracing" What do you mean by this sentence?

Response: Rephrased the sentence with explanation.

Page 3: "At times, a group of students become a very critic of religious beliefs. It becomes challenging to complete the assignments allotted to students with equal representation of female students where interaction must be quite open and need fluency in English" It is not clear what is the point you are trying to make by these sentences.

Response: Rephrased whole paragraph with the intent of clarity.

Page 3: "good Universities" How could you define good and bad universities? Response: Corrected and removed the sentence of “good universities”.

Page 5: "Religious rights wish to stay further in Australia" There is something missing in this phrase.

Response: Sorry it was by mistake. Rephrased whole sentence with the intent of clarity.

Page 7: ". Most of the 166(75.8%)" most of what ?

Response:

Corrected and rephrased the sentence

Page 11: "According to a recent, study, " grammar.

Response: Corrected

Reviewer #2:

Readable paper, plenty of interesting findings and an under-researched group.
Should have appeal for healthcare researchers and those interested in the Middle East.

The short title needs to have ‘Saudi Arabian’ in it.

Response: Thanks, included.

It is reasonably understandable on the whole, but I would recommend that a native English speaker review the next draft.

Response: We have got it edited by a professional editing service.

Further comment:
In Table 3, there is a ‘Statement’ column, but they are often phrased as questions. They need to be fixed one way or the other.

Response: Thanks for the observation, changed accordingly
P. 10 – ‘There is a lot of’ (cf there are many).

Response: Thanks, noted and corrected
p. 11 ‘Midgley W. (2009) reported Saudi students’ complained of being homesick as they couldn't keep their families with them [20].
So, do you think that the findings in your research could be a function of more family-friendly policies now? Other causes?

Response: Yes. We have explained this.

P. 12 (55.7%): do not need brackets here. You only need them when you use a number and you want to include the percentage as well: then you put the % in brackets. Response: Thanks, noted and sentence has been rephrased.
P. 13 ‘SA introduced English as a compulsory subject from early school teaching and announced it as official teaching language in all the Universities [33]’. So, is this a change from [when]?
‘Interestingly, 60.7% of participants mentioned they speak English at home with their families’. So, is this a change as well?
These two comments need further interpretation.

Response: We have added a sentence to clarify the situation.

Alzahrani (ref 16) does look like a PhD but it calls itself ‘Thesis submitted as a partial fulfilment to obtain the degree of Graduate Certificate in Research Methods’. Also, I cannot see a date on it. Can you clarify?
In the Discussion section, you state:
‘this is the first study regarding Medical students' experiences in Australia’, so you need to say this is the first survey of Saudi Arabian medical students … to differentiate it from, for example, Warren Midgley’s work.

Response: Reshaped the whole paragraph. Date added to Alzharani A (which is reference # 20 now)

Conclusion
‘Our findings revealed that Saudi medical students' psychosocial wellbeing, cultural adaptation, and academic success in Australia’
There is no main clause in this sentence – needs to be re-written. Furthermore, such a short conclusion does not capture your main findings. The paper should finish showing the ‘take home message’ for the readers.

Response: The Conclusion section has been rewritten keeping in view the expert advice of our reviewers

Stylistic comments
Abbreviations: eg ‘it’s’ cf ‘it has’
Paragraphing: It becomes challenging (new paragraph)
Lack of clarity: ‘faced fewer problems than their (the) senior students’
Word choice: the present study was investigated
rather embracing = ‘overwhelming’?
in their homes (in their home country?)
‘supported these girls to get admitted to medical schools’, cf ‘women’
‘by visualizing the better living facilities’ cf ‘by seeing’?
‘children mixing-up (mixing in) with Australian friends’
Table 2: ‘kids’ (children)
‘so 40% of our participants mentioned that they have never tried for any part-time job. The part-time jobs can be easily obtained in smaller cities & towns compared to big cities [30], and 14% of our study participants pointed out that when they required a part-time job, they got it without difficulty. Less than half of the students got the job when required but with some difficulty’
This is interesting and would benefit from disentangling: so, is is without difficulty or with some difficulty?
Avoiding sexist language
‘to speak out his point of view’, cf ‘their’.

There is still some very cognate research the authors could look at, for example, a paper published in 2012 (Clerehan et al.) in the International Nursing Review on Saudi Arabian nurses' experiences of studying Masters degrees in Australia. The method is different (interviews) and it has a small sample, but the findings have some commonalities (and differences, and I wonder if that is a function of time passing).

Response: The suggested reference has been added. It’s a nice qualitative study.

Table 1 and 2 appear to overlap.

Response: First table is showing general characteristics of our study participants while in second table we statistically analyzed our findings among different groups.
The two figures do not have captions and it's not clear if they are % or N.

Response: Captions updated in both the figures, in both the number of students is shown not the percentage

 6. PLOS authors have the option to publish the peer review history of their article ([what does this mean?](https://journals.plos.org/plosone/s/editorial-and-peer-review-process#loc-peer-review-history)). If published, this will include your full peer review and any attached files.

If you choose “no”, your identity will remain anonymous but your review may still be made public.

**Do you want your identity to be public for this peer review?** For information about this choice, including consent withdrawal, please see our [Privacy Policy](https://www.plos.org/privacy-policy).

Reviewer #1: No

Reviewer #2: **Yes:** R Clerehan

[NOTE: If reviewer comments were submitted as an attachment file, they will be attached to this email and accessible via the submission site. Please log into your account, locate the manuscript record, and check for the action link "View Attachments". If this link does not appear, there are no attachment files.]

While revising your submission, please upload your figure files to the Preflight Analysis and Conversion Engine (PACE) digital diagnostic tool, <https://pacev2.apexcovantage.com/>. PACE helps ensure that figures meet PLOS requirements. To use PACE, you must first register as a user. Registration is free. Then, login and navigate to the UPLOAD tab, where you will find detailed instructions on how to use the tool. If you encounter any issues or have any questions when using PACE, please email PLOS at [figures@plos.org](mailto:figures@plos.org). Please note that Supporting Information files do not need this step.
