# Supplementary material for: Psychosocial, cultural, and academic challenges to Saudi Arabian students in Australia
Source: PLoS One. 2022 Jan 31;17(1):e0262585. doi: 10.1371/journal.pone.0262585 (PMC8803179; doi:10.1371/journal.pone.0262585)
Supplement: Attachment — Submitted filename: Response to reviewers.docx [file pone.0262585.s002.docx]

PONE-D-21-00081R1

Psychosocial, cultural, and academic challenges to Saudi Arabian students in Australia

PLOS ONE

Journal Requirements:

Please review your reference list to ensure that it is complete and correct. If you have cited papers that have been retracted, please include the rationale for doing so in the manuscript text, or remove these references and replace them with relevant current references. Any changes to the reference list should be mentioned in the rebuttal letter that accompanies your revised manuscript. If you need to cite a retracted article, indicate the article’s retracted status in the References list and also include a citation and full reference for the retraction notice.

Reply:

We have checked the reference list and several references have been corrected. No reference has been removed or added.

Reviewers' comments:

Reviewer's Responses to Questions

**Comments to the Author**

1. If the authors have adequately addressed your comments raised in a previous round of review and you feel that this manuscript is now acceptable for publication, you may indicate that here to bypass the "Comments to the Author" section, enter your conflict of interest statement in the “Confidential to Editor” section, and submit your "Accept" recommendation.

Reviewer #1: All comments have been addressed

Reviewer #3: (No Response)

2. Is the manuscript technically sound, and do the data support the conclusions?

The manuscript must describe a technically sound piece of scientific research with data that supports the conclusions. Experiments must have been conducted rigorously, with appropriate controls, replication, and sample sizes. The conclusions must be drawn appropriately based on the data presented.

Reviewer #1: Yes

Reviewer #3: Yes

3. Has the statistical analysis been performed appropriately and rigorously?

Reviewer #1: Yes

Reviewer #3: Yes

4. Have the authors made all data underlying the findings in their manuscript fully available?

The [PLOS Data policy](http://www.plosone.org/static/policies.action#sharing) requires authors to make all data underlying the findings described in their manuscript fully available without restriction, with rare exception (please refer to the Data Availability Statement in the manuscript PDF file). The data should be provided as part of the manuscript or its supporting information, or deposited to a public repository. For example, in addition to summary statistics, the data points behind means, medians and variance measures should be available. If there are restrictions on publicly sharing data—e.g. participant privacy or use of data from a third party—those must be specified.

Reviewer #1: Yes

Reviewer #3: Yes

5. Is the manuscript presented in an intelligible fashion and written in standard English?

PLOS ONE does not copyedit accepted manuscripts, so the language in submitted articles must be clear, correct, and unambiguous. Any typographical or grammatical errors should be corrected at revision, so please note any specific errors here.

Reviewer #1: Yes

Reviewer #3: No

6. Review Comments to the Author

Please use the space provided to explain your answers to the questions above. You may also include additional comments for the author, including concerns about dual publication, research ethics, or publication ethics. (Please upload your review as an attachment if it exceeds 20,000 characters)

Reviewer #1: (No Response)

Reviewer #3: In this study, Jamil et al. analyze the psychological, cultural and academic challenges Saudi Arabian students face while studying in Australia. Of the 219 students included in this analysis, we observe majority respondents to be postgraduates, and well-adjusted to the Australian system and culture.

This revised manuscript is significantly better, and authors have done a good job in responding to majority of prior queries. However, I do have a few minor comments:

1) The manuscript could be significantly improved by a copy editor, or having the document proof read as there are a number of stylistic errors in the text.

Reply:

The manuscript has been proofread and several sentences rephrased, and stylistic errors have been removed.

Abstract:

2) In the results portion of the abstract, I would suggest authors to mention numerical values instead of writing 'most' or 'few' in lines 3-6 of results. This would help ascertain the actual magnitude of students satisfied with their academic performance and adjustment in Australia.

Reply:

Thank you for the suggestion. The numerical values have been mentioned as suggested.

3) Last four lines of Results in Abstract "Students' responses regarding responded to a question, "Do you wish to stay in Australia after completion of your studies?", This should be removed to only include the results. Hence authors should instead write “ 77 (35.1%), 119(54.3%), and 23(10.5%) students indicated that they wished to stay in Australia only until completion of their studies, temporarily and permanently, respectively”

Reply:

Thank you for the suggestion. The sentence has been modified as suggested.

Introduction

1) Line 4: "The Ministry of Education of the Kingdom of Saudi Arabia (KSA) sponsored 174333 students for abroad education” This should be written as “The Ministry of Education of the Kingdom of Saudi Arabia (KSA) sponsored 174333 students for education abroad”.

Reply:

Thank you for the suggestion. The sentence has been modified as suggested.

2) As raised earlier, the line “In the case of interactive lectures, the situation becomes rather embarrassing when it becomes difficult for them to take participate in active discussions and question answer- sessions in classes and ward rounds” does not sit so well with me. It may not be embarrassing, rather difficult to partake in fruitful academic discussions due to language barrier, which may lead to lack of participation. I would suggest the authors to revise this sentence.

Reply:

Thank you for the suggestion. The sentence has been modified as suggested.

3) “International students, especially medical undergraduates, and postgraduates, are among the most successful students in their home countries [6]. This could be because of the better training opportunities and advanced technology available at the universities of the developed countries’ universities.”

This is a bit unclear to me. Do the authors mean developed countries such as Australia or the home country KSA? This should instead be written as international students being successful in their home institutions, who then in search of better training opportunities wish to go abroad. Right now it seems as if the authors are talking about students having better training opportunities in their home country, that’s why they are successful. If this is the case then why would they wish to go abroad? Some clarity would be good to make a strong introduction.

Reply:

Thank you for the suggestion. The sentence has been modified as suggested.

4) "While in Australia teaching is all mixed in Australia”. Suggest to use the word co-education here

Reply:

Thank you for the suggestion. The sentence has been modified as suggested.

Results

1) How many Australian institutes was the data obtained from?

Reply:

Data were obtained from 42 Australian institutes (This has been included in the results).

Discussion

1) "The body of the lliterature , indicated” , suggest this to instead write as “current literature indicates”

Reply:

Thank you for the suggestion. The sentence has been modified as suggested.

2) Future recommendations point 1 "Arabic is a very powerful language, and most of the time, even teacher- student interaction is in the native Arabic language. So, the teachers ensure that , the discussion medium should be only English during classroom discussions and explanations.” Suggest authors to revise this. Difficult to follow the sentence.

Reply:

Thank you for the suggestion. The sentence has been modified as suggested.

 7. PLOS authors have the option to publish the peer review history of their article ([what does this mean?](https://journals.plos.org/plosone/s/editorial-and-peer-review-process#loc-peer-review-history)). If published, this will include your full peer review and any attached files.

If you choose "no", your identity will remain anonymous but your review may still be made public.

**Do you want your identity to be public for this peer review?** For information about this choice, including consent withdrawal, please see our [Privacy Policy](https://www.plos.org/privacy-policy).

Reviewer #1: No

Reviewer #3: No
