# Supplementary material for: Psychosocial, cultural, and academic challenges to Saudi Arabian students in Australia
Source: PLoS One. 2022 Jan 31;17(1):e0262585. doi: 10.1371/journal.pone.0262585 (PMC8803179; doi:10.1371/journal.pone.0262585)
Supplement: Attachment — Submitted filename: Response to reveiwers.docx [file pone.0262585.s003.docx]

PONE-D-21-00081R2

Psychosocial, cultural, and academic challenges to Saudi Arabian students in Australia

Journal Requirements:

Please review your reference list to ensure that it is complete and correct. If you have cited papers that have been retracted, please include the rationale for doing so in the manuscript text, or remove these references and replace them with relevant current references. Any changes to the reference list should be mentioned in the rebuttal letter that accompanies your revised manuscript. If you need to cite a retracted article, indicate the article’s retracted status in the References list and also include a citation and full reference for the retraction notice.

[Note: HTML markup is below. Please do not edit.]

Reply:

We have removed four references because of duplication. We have avoided including any retracted article.

Reviewers' comments:

Reviewer's Responses to Questions

**Comments to the Author**

1. If the authors have adequately addressed your comments raised in a previous round of review and you feel that this manuscript is now acceptable for publication, you may indicate that here to bypass the "Comments to the Author" section, enter your conflict of interest statement in the “Confidential to Editor” section, and submit your "Accept" recommendation.

Reviewer #3: All comments have been addressed

Reviewer #4: (No Response)

2. Is the manuscript technically sound, and do the data support the conclusions?

The manuscript must describe a technically sound piece of scientific research with data that supports the conclusions. Experiments must have been conducted rigorously, with appropriate controls, replication, and sample sizes. The conclusions must be drawn appropriately based on the data presented.

Reviewer #3: Yes

Reviewer #4: Partly

3. Has the statistical analysis been performed appropriately and rigorously?

Reviewer #3: Yes

Reviewer #4: Yes

4. Have the authors made all data underlying the findings in their manuscript fully available?

The [PLOS Data policy](http://www.plosone.org/static/policies.action#sharing) requires authors to make all data underlying the findings described in their manuscript fully available without restriction, with rare exception (please refer to the Data Availability Statement in the manuscript PDF file). The data should be provided as part of the manuscript or its supporting information, or deposited to a public repository. For example, in addition to summary statistics, the data points behind means, medians and variance measures should be available. If there are restrictions on publicly sharing data—e.g. participant privacy or use of data from a third party—those must be specified.

Reviewer #3: Yes

Reviewer #4: Yes

5. Is the manuscript presented in an intelligible fashion and written in standard English?

PLOS ONE does not copyedit accepted manuscripts, so the language in submitted articles must be clear, correct, and unambiguous. Any typographical or grammatical errors should be corrected at revision, so please note any specific errors here.

Reviewer #3: Yes

Reviewer #4: Yes

6. Review Comments to the Author

Please use the space provided to explain your answers to the questions above. You may also include additional comments for the author, including concerns about dual publication, research ethics, or publication ethics. (Please upload your review as an attachment if it exceeds 20,000 characters)

Reviewer #3: (No Response)

Reviewer #4: I had the opportunity to review the revised manuscript, “Psychosocial, cultural, and academic challenges to Saudi Arabian students in Australia”, for possible publication in the Journal of PLOS ONE. The genesis of the presented study is the fact that studying abroad is associated with psychological, cultural, and academic challenges. In their paper, the authors describe the subjective experience of saudi students of medical related professions in Australia. The authors found that saudi students were overall satisfied with their experience in Australia though this is difficult be generalized to other samples or compared with other results. kindly find my few comments enumerated below.

1- The authors stated that the study was conducted during the year of 2019. The authors may specify the exact timeframe over which the study was conducted, and data were collected as the temporal factors may have a significant effect on participants attitudes.

Reply: It was conducted from March 15 to June 15, 2019. We have incorporated this in the manuscript.

2- Snowball sampling technique was employed to reach out to participants. Could the number of directly contacted participants be specified?

Reply: We contacted 50 students directly. This statement has been included in the methodology of the revised manuscript.

3- “The questionnaire was formulated in English with the help of already published studies”. Could the authors clarify the rationale behind employing this particular questionnaire, and how they insured the validity and the reliability of the questionnaire?

Reply: We found that these questions thoroughly evaluate the psychosocial, cultural, and academic challenges Saudi Arabian students face in Australia. Therefore, these questions were used in the present study. The content and construct validity were ensured by evaluating these questions by two senior professors and a medical educationist proficient in English. Moreover, the questionnaire was sent to a few students to check its comprehension and later modified after receiving content experts’ and students’ suggestions. The questionnaire reliability was calculated by Cronbach’s alpha, and its value was 0.82. We have included the above information in the revised manuscript.

4- Based on the questionnaire responses, how the participants were considered satisfied or not satisfied with their experience? This may be clarified within the methodology section. In other words, when satisfaction was considered as a result?

Reply: Thank you very much for pointing out this. We have incorporated this point in the methodology. "If more than 60% of students selected options highly satisfactory and satisfactory or unsatisfactory and highly unsatisfactory, were considered satisfied and unsatisfied, respectively.”

5- It would be valuable if the Tables and Figures were understandable as stand-alone. I believe that Tables and Figures need more clear descriptive titles.

Reply: Thanks for pointing out this. We have modified the titles of all tables and figures.

6- Unmentioned limitation of this study is the use of subjective self-reported measure and associated biases. For instance, acquiescence bias, where people tend to agree on questionnaire, may skew the result form the truth.

Reply: Thank you for pointing out this. We have mentioned a new sentence in the limitation. “A subjective self-reported measure and accompanying biases such as acquiescence and dissent biases are also possible.”

7- The conclusions need to be supported by the presented data. The first two sentences in the conclusion section cannot be extrapolated from the presented result.

Reply: Thank you for pointing out this. We have removed the first two sentences from the conclusion.

8- The reference list has some duplicate references. Please omit any duplicate.

Reply: Thank you for pointing out this. We are sorry for this mistake. All duplicated references have been removed.

7. PLOS authors have the option to publish the peer review history of their article ([what does this mean?](https://journals.plos.org/plosone/s/editorial-and-peer-review-process#loc-peer-review-history)). If published, this will include your full peer review and any attached files.

If you choose "no", your identity will remain anonymous but your review may still be made public.

**Do you want your identity to be public for this peer review?** For information about this choice, including consent withdrawal, please see our [Privacy Policy](https://www.plos.org/privacy-policy).

Reviewer #3: No

Reviewer #4: No

[NOTE: If reviewer comments were submitted as an attachment file, they will be attached to this email and accessible via the submission site. Please log into your account, locate the manuscript record, and check for the action link "View Attachments". If this link does not appear, there are no attachment files.]

While revising your submission, please upload your figure files to the Preflight Analysis and Conversion Engine (PACE) digital diagnostic tool, <https://pacev2.apexcovantage.com/>. PACE helps ensure that figures meet PLOS requirements. To use PACE, you must first register as a user. Registration is free. Then, login and navigate to the UPLOAD tab, where you will find detailed instructions on how to use the tool. If you encounter any issues or have any questions when using PACE, please email PLOS at [figures@plos.org](mailto:figures@plos.org). Please note that Supporting Information files do not need this step.
